# Acute compartment syndrome caused by hematoma with minor trauma in hemodialysis patients: Two case reports

**DOI:** 10.1016/j.ijscr.2023.108594

**Published:** 2023-08-04

**Authors:** Shinsuke Morisaki, Yusuke Kobayashi, Shinji Tsuchida, Kenji Takahashi

**Affiliations:** a Department of Orthopaedics, Saiseikai Shiga Hospital, Ohashi 2-4-1, Ritto, Shiga 520-3046, Japan; b Department of Orthopaedics, Kyoto Prefectural University of Medicine, Kawaramachi Hirokoji, Kamigyo-ku, Kyoto 602-8566, Japan

**Keywords:** Compartment syndrome, Hemodialysis, Forearm, Minor trauma

## Abstract

**Introduction:**

Acute compartment syndrome is a condition that requires an immediate diagnosis and surgical management. Compartment syndrome related to hematoma caused by minor trauma in hemodialysis patients is rarely reported.

**Case presentation:**

We present two cases of hemodialysis patients diagnosed with compartment syndrome of the forearm due to hematoma caused by the disruption of blood vessels after a minor trauma. The removal of the hematoma and fasciotomy with adequate skin care significantly improved soft tissue heeling with no functional impairment.

**Discussion:**

A long-term history of hemodialysis may increase the vascular vulnerability and have the potential risk of disruption by minor trauma. When the blood vessel is disrupted, a hematoma is formed and necessitating emergent surgical intervention.

**Conclusion:**

Surgeons should be aware of the potential risk of damage to blood vessels with minor trauma that results in the formation of a hematoma and compartment syndrome in hemodialysis patients.

## Highlights

• Compartment syndrome related to hematoma caused by minor trauma in hemodialysis patients is rarely reported.
• Disruption of blood vessels may cause hematoma, thereby necessitating emergent surgical intervention.
• Surgeons should be aware of the potential risk of damage to a collateral artery with minor trauma that results in the formation of a hematoma and compartment syndrome in hemodialysis patients.

## 1 Introduction

Compartment syndrome is a condition caused by increasing pressure within tightly bound myofascial compartments [1,2]. Various causes have been reported for compartment syndrome such as crush injuries and high-energy fracture [3,4]. However, compartment syndrome can also occur with low-energy trauma, although it is relatively rare.

We report two cases of acute compartment syndrome associated with minor trauma in patients who were undergoing hemodialysis. A slight trauma caused disruption in the blood vessels of the forearm, thereby leading to the formation of a hematoma, which required emergent operation.

Acute compartment syndrome has previously been reported in patients undergoing hemodialysis in whom bleeding after the puncture of a hemodialysis fistula caused a hematoma [5,6]. However, our cases were not associated with the puncture of a fistula. The case reports adhere to SCARE criteria [7].

## 2 Case 1

**Fig. 1:**
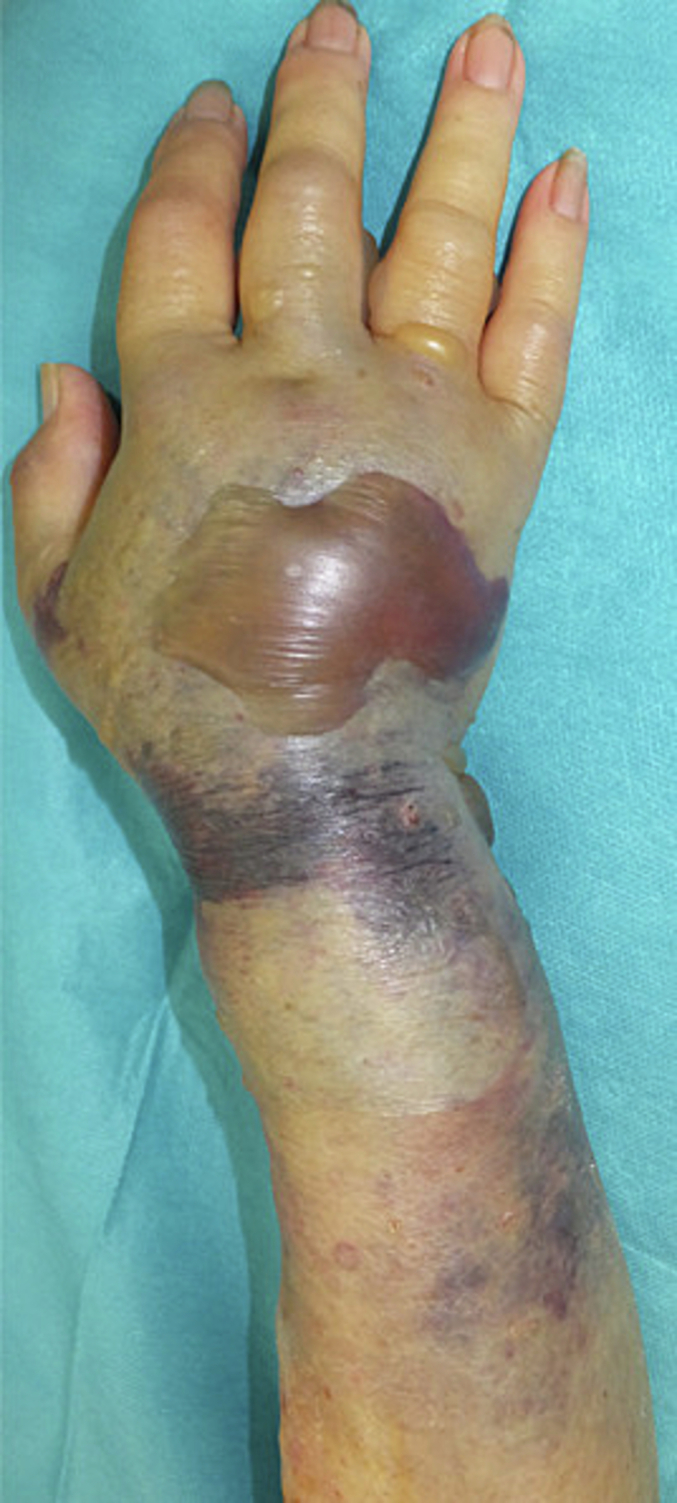
A 60-year-old woman, who had a 35-year history of hemodialysis, presented with swelling and blister formation from her right forearm to the dorsal hand 24 h after falling and hitting the ground.

**Fig. 2:**
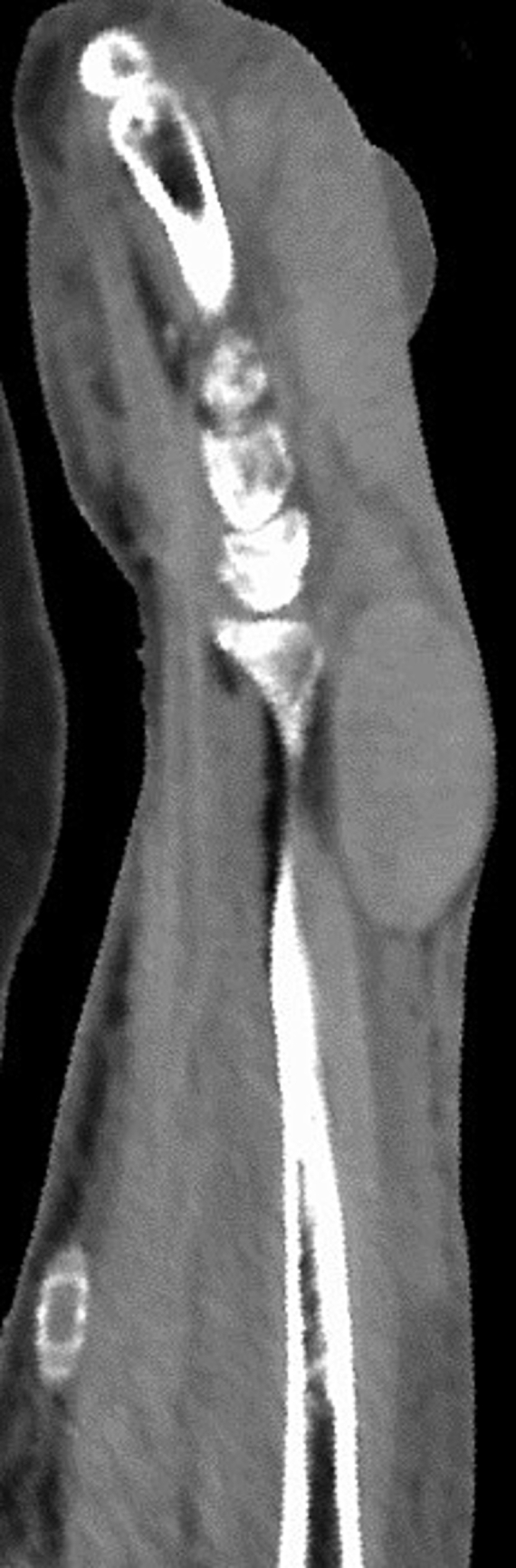
Computed tomography (CT) imaging shows a hematoma on the dorsal forearm.

**Fig. 3:**
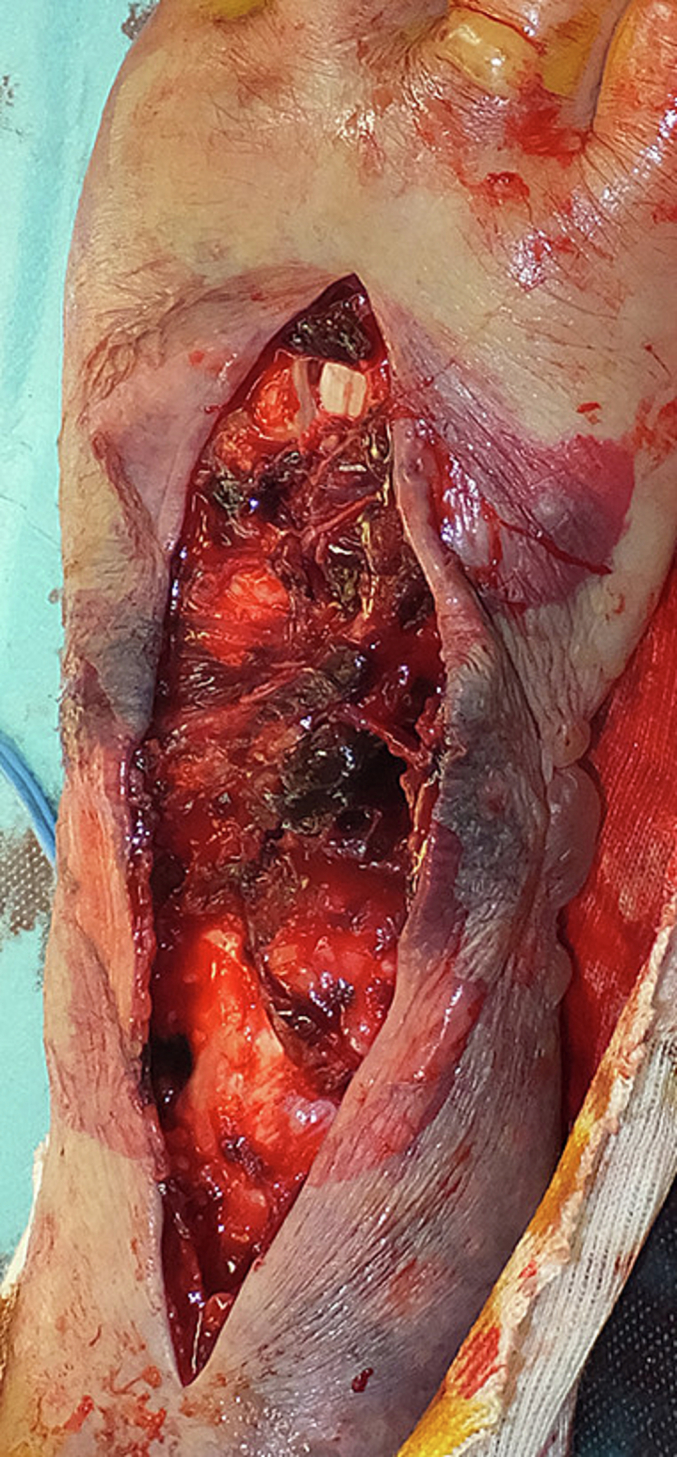
During surgery, active bleeding from the collateral artery was noted.

**Fig. 4:**
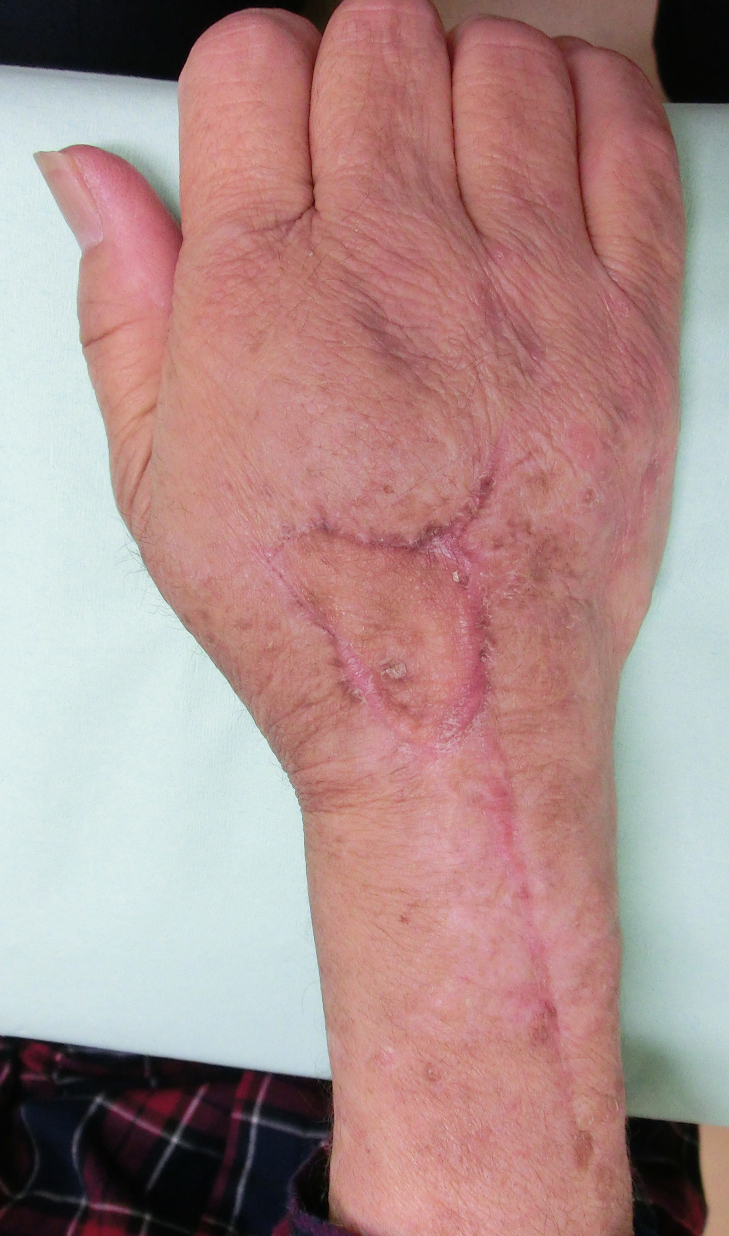
At 6 months postoperation, the wound has healed with no limited range of motion of the wrist and the fingers.

A 60-year-old woman with end-stage renal failure secondary to glomerulonephritis had a right radiocephalic anterior fistula for 35 years. She presented to our emergency room with swelling of the right forearm caused by falling and hitting the ground with her right hand. An examination showed swelling from her forearm to dorsal hand without a visible fracture on the x-ray image, no abnormalities in sensation, and an active range of motion. At that time, she returned home because the possibility of compartment syndrome seemed unlikely. The next day, she revisited the emergency room because of increased pain and progressive swelling of the forearm. The examination revealed tense swelling with severe ecchymosis and blistering of the skin around the forearm to the dorsal hand with limited range of motion of the fingers (Fig. 1). Computed tomography (CT) revealed a hematoma on the dorsal forearm distal from the site of the fistula (Fig. 2). Her activated partial thromboplastin time (APTT) ratio was normal, although she was taking anticoagulants. Compartment syndrome due to hematoma was diagnosed. Emergency exploration was undertaken via a dorsal incision (Fig. 3). There was a hematoma as well as the swelling of extensor muscles proximal to the hematoma. The bleeding was from an arterial branch and was stopped with bipolar coagulation forceps, and fasciotomy was performed around the hematoma. The wound of forearm to the dorsal hand was debrided and a skin defect of 5 × 5 cm^2^ was on the dorsal hand. Therefore, a negative-pressure wound dressing was administered. Seven days after injury, a full-thickness skin graft was applied. The wound was fully healed at 2 months. At 6 months after the operation, the patient had no limited range of motion of the wrist and fingers or neurological deficits (Fig. 4). The pain score on the visual analog scale (VAS; range, 0–10) was 0. The Quick Disabilities of the Arm, Shoulder, and Hand Questionnaire (quick DASH) score was 11.4.

## 3 Case 2

**Fig. 5:**
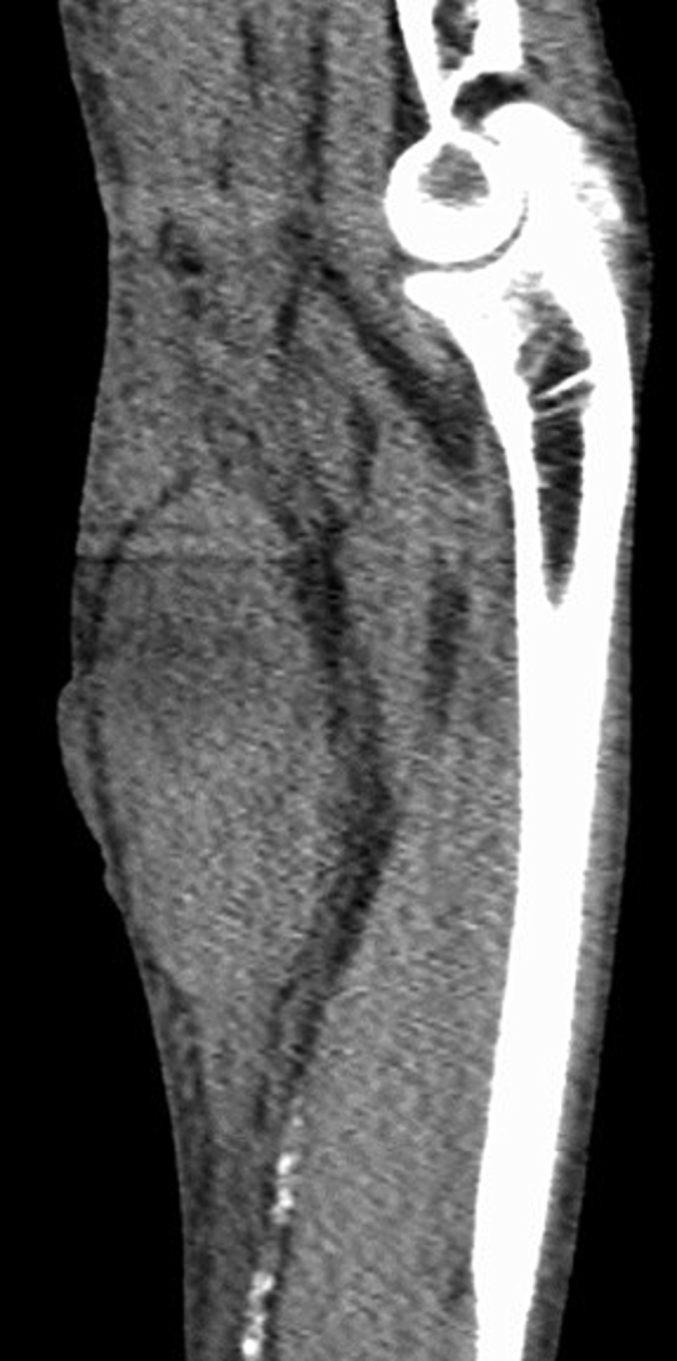
CT imaging shows a hematoma on the volar forearm.

A 73-year-old man with end-stage renal failure secondary to type 2 diabetes mellitus had a 13-year history of hemodialysis with a left radiocephalic arteriovenous fistula. He presented to our emergency room because of swelling of the left volar forearm that rapidly developed 3 h after hitting his forearm on a handrail when he transferred to a wheelchair. An examination revealed a tense swollen forearm with severe ecchymosis and blistering of the skin. His APTT ratio was normal, although he was taking anticoagulants. X-ray imaging revealed no fractures of the forearm. CT showed a hematoma on the volar side of the forearm (Fig. 5). Limited range of motion of the fingers due to increased swelling and pain was evident; therefore, acute compartment syndrome was diagnosed. Emergency exploration was undertaken via a volar incision. During the operation, 260 mL of intermuscular and subcutaneous hematoma were evacuated and fasciotomy was performed around the hematoma. The bleeding was stopped with bipolar coagulation forceps. The wound of forearm was debrided and closed using the shoelace technique. The wound was closed 14 days after swelling of the forearm had decreased. The wound was fully healed at 2 months. Six months after the operation, the patient had no limited range of motion of the elbow or wrist and had no neurological deficits. The pain score on the VAS was 0 and the quick DASH score was 0.

## 4 Discussion

We presented two patients with a long history of hemodialysis who had acute compartment syndrome in the forearm and hand caused by minor trauma that resulted in the formation of hematoma. This phenomenon has never been reported.

Compartment syndrome develops from any condition that increases the compartmental content without a commensurate increase in the size of myofascial envelope [3]. The most common causes of compartment syndrome are related to traumatic injury such as a fracture or dislocation.

However, compartment syndrome from minor trauma has also been reported, although it is relatively rare. Such cases may have potential risks such as medical comorbidities associated with abnormal bleeding diatheses (e.g., the use of coagulants or clotting disorders), volume resuscitation, altered mental status, or neurologic compromise that diminishes sensitivity or sensibility in a limb [4].

A previous study has reported seven cases of acute compartment syndromes caused by minor trauma resulting from anticoagulant treatment [8]. Other reports have described cases of acute forearm compartment syndrome associated with dialysis access fistula bleeding after a puncture [5]. In this situation, vascular perforation and heparin overdoses were risk factors. Investigators in another study reported five cases of forearm compartment syndrome, after hemodialysis access fistula puncture [6].

The patients in our case reports had also been on hemodialysis, but fistula puncture was not associated with the occurrence of compartment syndrome. In both of our patients, minor trauma such as hitting the affected limb against an object but without a fracture caused the rupture of the blood vessel, resulting in the formation of a hematoma. Both patients had a long history of dialysis because of chronic kidney disease and had received anticoagulants; however, their APTT was not prolonged and was controlled adequately. Therefore, anticoagulants may not be a main cause of bleeding.

For a diagnosis of acute compartment syndrome, an examination of the intermuscular pressure by needle has been suggested [2]. However, in our patients, the hematoma expanded under the skin with disruption of the intermuscular compartment. Furthermore, skin color changes were visible. Therefore, the patients clearly had compartment syndrome and needle pressure assessment was skipped because of the risk of the progression in bleeding.

For treatment, removing the hematoma and fasciotomy were effective. When a skin defect existed, a skin graft was necessary, as in the first patient. The early detection of compartment syndrome and early control of bleeding can help reduce further damage to the skin and soft tissue.

In conclusion, patients who have a long-term history of hemodialysis may have a risk of acute compartment syndrome resulting from minor trauma that causes a hematoma. Explaining the risks of compartment syndrome to a patient is important. When progressive swelling and blisters occur, suggesting a visit to an emergency department and early surgical treatment are necessary to prevent functional impairment.

## Ethical approval

Ethical approval for this study (Ethical Committee #559) was provided by the Ethical Committee of Saiseikai Shiga Hospital, Shiga, Japan on 30 January 2023.

## Funding

The authors received no financial support for the preparation, research, authorship, and/or publication of this manuscript.

## CRediT authorship contribution statement

Morisaki S wrote the manuscript and prepared the figures. Morisaki S was the main surgeon, and is a specialist in hand surgery. Kobayashi Y, Tsuchida S and Takahashi K contributed to the conception and design of the study and critically revised the manuscript. All authors read and approved the final manuscript.

## Guarantor

Shinsuke Morisaki.

## Research registration number

Not applicable.

## Informed consent

Written informed consent was obtained from the patient for publication and any accompanying images. A copy of the written consent is available for review by the Editor-in-Chief of this journal on request.

## Declaration of competing interest

The authors do not have any potential conflicts of interest with respect to this manuscript.
